# The Potential Role of Graphene in Developing the Next Generation of Endomaterials

**DOI:** 10.1155/2016/3180954

**Published:** 2016-11-29

**Authors:** Nikolaos Patelis, Demetrios Moris, Sean Matheiken, Chris Klonaris

**Affiliations:** ^1^First Department of Surgery, Vascular Unit, Laiko General Hospital, National and Kapodistrian University of Athens, Athens, Greece; ^2^Lerner Research Institute, Cleveland Clinic Foundation, Cleveland, OH, USA; ^3^Vascular Unit, Royal Free Hospital, London, UK

## Abstract

Graphene is the first 2-dimensional material and possesses a plethora of original properties. Graphene and its derivatives have exhibited a great potential in a number of fields, both medical and nonmedical. The aim of this review is to set the theoretical basis for further research in developing graphene-based endovascular materials. An extensive search was performed in medical and bioengineering literature. Published data on other carbon materials, as well as limited data from medical use of graphene, are promising. Graphene is a promising future material for developing novel endovascular materials. Certain issues as biocompatibility, biotoxicity, and biostability should be explored further.

## 1. Introduction

Material specifications and techniques are critical for the evolution of endovascular surgery.

Graphene is a two-dimensional carbon allotrope comprised of sp^2^-bonded carbon atoms in a sheet-like arrangement. Its unique structural, chemical, thermal, and other properties [1–3] have been demonstrated in many fields [4–15]. Graphene is produced through various processes, the most common of which are mechanical exfoliation and chemical vapor deposition [16]. Its derivatives include graphene oxide (GO).

The use of GO as a delivery substrate of water-insoluble cancer medication was first reported in 2008. The properties of graphene and its derivatives have demonstrated immense biomedical applicability [12, 14, 15, 17–23]. This literature review summarises the existing bibliography on the potential of graphene and its derivatives to change current endovascular materials and techniques.

## 2. Special Characteristics of Graphene

### 2.1. Thrombogenicity

*In vitro* studies by Paul and Sharma in 2011 reported that graphene, when in contact with blood, does not absorb activated C3 (C3a), indicative of complement activation not taking place [24]. In a similar manner, serum Platelet Factor 4 (PF4) levels can be used to assess the level of platelet activation; PF4 levels did not change significantly when platelets were in contact with graphene. Both of these findings suggested that the tested graphene sample is biocompatible.

The ratio of adsorbed serum fibrinogen to albumin (FAR) indicates both the biocompatibility of materials in contact with blood and the degree of platelet activation and clot formation in a proportionate manner [25]. Graphene coating on nitinol (Gr–NiTi) exhibits lower FAR compared to NiTi* in vivo*, supporting the biocompatibility of graphene [26, 27].

Fibrin, the first step towards thrombus formation, forms when electrons transfer from fibrinogen molecules to a biomaterial. At room temperature, graphene sheets do not demonstrate significant electron transfer; therefore, graphene does not trigger conversion of fibrin to fibrinogen and the consequent thrombosis. This ability of graphene to not induce clot formation could be expanded by drug-eluting graphene scaffolds. The lower thrombogenicity of graphene could potentially decrease stent occlusion.

Graphene is rendered a suitable drug-delivery substrate due to the presence of reactive functional groups and localised *π*-electrons, promoting *π*–*π* bonds with other molecules [28, 29]. Weaver et al. developed a GO-based delivery system controlled by electrical impulses to release dexamethasone into the bloodstream [30]. Other studies have reported the transport and release of antithrombotic agents by graphene [31, 32].

Lee et al. reported that the conjugate of graphene and unfractionated heparin resulted in enhanced anti-Xa activity approximately 30-fold higher than a solitary GO coating [31].

Xue et al. used graphene coating* in vitro* as a substrate for integrating antithrombotic enzymic systems [33].

### 2.2. Biotoxicity and Biocompatibility

Chemical vapor deposition during graphene development increases caspase-3 activation, the lactate dehydrogenase release, and the generation of reactive oxygen species in neural pheochromocytoma-derived PC12 cells [34].

Wang et al. reported that GO demonstrated a cytotoxic effect on human fibroblast cells in blood concentrations exceeding 50 mg/L [35]. Hu et al. reported that GO slightly reduced A549 cells proliferation rate at concentrations higher than 85 mg/L, without resulting in cell death [36]. The same cell lines were remarkably affected by the cytotoxicity of reduced-GO (r-GO) after treatment by hydrazine hydrate. The cytotoxic effect of the reactive oxygen species (ROS) released by the graphene surface has been reported to affect A549 cells and neural pheochromocytoma-derived PC12 cells [37]. Akhavan et al. reported that r-GO nanoribbons are cytotoxic on human mesenchymal stem cells (hMSCs) in concentrations as low as 10 *μ*g/mL in contrast to r-GO sheets that become toxic in higher concentrations (>100 *μ*g/mL) [38]. These results were demonstrated at a short incubation period of 1 hour. Another publication by Akhavan et al. reports that r-GO nanoribbons of average lateral dimensions of 11 ± 4 nm could penetrate into hMSCs and cause significant DNA fragmentation and chromosomal aberrations even at low doses between 0.1 and 1.0 *μ*g/mL after 1 hour [39]. On the contrary, r-GO sheets showed no significant cytotoxicity even at 10-fold doses.

On the contrary, GO covered in a biocompatible coating showed low* in vitro* cytotoxicity to various cell lines, even at concentrations reaching 100 mg/L [22, 40].

Animal studies on* in vivo* cytotoxicity of graphene have reported significant GO accumulation in lungs of rats and mice after intravenous GO injection. Pulmonary toxicity was dose-dependent and obvious at GO doses ≥10 mg/kg [35, 41]. Other studies support the accumulation of graphene molecules not only in lungs, but also in spleen, testis, kidney, thymus, heart, liver, and brain [42].

Radiolabeled intravenously administered polyethylene glycol covered GO (GO-PEG) in an animal model accumulates mainly in the reticuloendothelial system and lung [43]. Radiolabeled GO-PEG administered over a 3-month period at a dose reaching 20 mg/kg was gradually excreted with insignificant systemic cytotoxicity.

A study of GO-PEG nanoribbons in cancer cell imaging and photothermal therapy concluded that the threshold of 1 *μ*g/mL causes <11% cell destruction and <7% DNA fragmentation and therefore it should be considered a safe dose [44]. A similar threshold has been suggested in another publication concluding that the* in vivo* dose of 1 mg graphene per kilogram of the animal model's body weight results in the same cytotoxic effect as 10 *μ*g/mL dose* in vitro, possibly due to the in vivo *hard corona and dilution [42]. High-dose injections (>200 *μ*g) or graphene concentrations higher than 100 *μ*g/mL demonstrate strong cytotoxic effects resulting in >72% cell death and >29% DNA fragmentation.

The biocompatibility of graphene is questioned by Monaco and Giugliano, due to the limited number of studies on different graphene derivatives [45]. The properties of each graphene form could induce various toxicological systemic responses and require further detailed research, similar to the work of Hashemi et al., who studied the toxicity of three r-GO sheets on spermatozoa [42]. In this study, the cytotoxic effect of the r-GO surface on moving cells is based primarily on physical trapping of the cells and secondarily on adenosine triphosphate (ATP) depletion, DNA fragmentation of the cells, and ROS generation. This cytotoxic effect was reported to be dose- and time-dependent, as well as dependent on the r-GO form used, with green tea polyphenols reduced-GO (GTP-r-GO) being the least toxic compared to hydrazine- or hydrothermal-reduced-GO.

Cytotoxicity of graphene sheets on reproduction and gestation of mammals of both genders has been reported by Akhavan et al. [46]. This toxicity is due to the same mechanisms as those described in the work of Hashemi et al. and is dose-dependent.

Podila et al. demonstrated the vascular biocompatibility in an animal model by finding insignificant* in vitro* toxicity upon smooth muscle cells and aortic endothelium [26, 27]. Aligned with this theory of enhanced biocompatibility, Mikhalovska et al. reported the hemocompatibility of hydrophobic carbon-coated metals to be higher than that of bare metal and these findings were supported by other studies [47, 48].

### 2.3. Antibacterial Effects

Two studies report a substantial loss in bacterial cell viability in contact with either GO or r-GO surfaces orientated such that a great number of exposed material edges exist [49].* E. coli *and* S. aureus *exposed to graphene derivatives sustained substantial viability loss. This was more prominent when exposed to r-GO, as sharper edges of the r-GO particles compared to other derivatives cause increased cytotoxicity.* S. aureus *and other Gram-positive bacteria were affected more severely, probably as the contact with the material surface induced membrane damage and efflux of cytoplasma [50, 51]. In partial contrast with these findings, Hu et al. [36] reported that* E. coli cells in contact *with GO and r-GO showed significant loss of viability, but GO surfaces demonstrated better results. Once more, the toxicity mechanism is considered to be bacterial membrane damage caused by material edges; the different results might be explained by the GO production method. Vacuum filtration results in material particles lying entirely flat with only a small number of material edges oriented perpendicularly.

In complete contrast, a study has suggested that there is no cytotoxic effect to bacteria in contact with GO and reported* E. coli* growth rates 3-fold higher with preferential attachment to areas with dense allocation of GO particles [52]. Vacuum filtration could again be the cause of these findings.

The antibacterial properties of GO or r-GO films in combination with a variety of other known antibacterial substances have also been studied. Lactoferrin and chitosan on a GO or r-Go composite film showed significant cytotoxicity against* E. coli* [53]. Other studies exist involving GO composite sheets with attached polyvinyl-N-carbazole, a polymer with antibacterial characteristics [54, 55]. The antibacterial activity of GO-polyvinyl-N-carbazole against* E. coli, C. metallidurans*,* B. subtilis*, and* R. opacus* was reported to be higher than the activity of unmodified GO surfaces, especially against Gram-positive bacteria. Despite the observed antibacterial activity of GO-polyvinyl-N-carbazole, there was insignificant cytotoxicity towards eukaryotic cells.

Antibacterial activity and stimulation of human cell growth on other GO-polymer composites have been studied and a composite of GO and poly-L-lysine (GO-PLL) showed potent cytotoxic effect on* E. coli* [56]. Diazonium salt covalently attached to GO-PLL led to further decrease in cell attachment and increased bacterial cell death.

Krishnamoorthy et al. found that r-GO minimum inhibitory concentration (MIC) was lower for Gram-negative bacteria antibacterial activity by using r-GO sheets, probably due to the thinner peptidoglycan layer of these organisms [57].

Only one publication reports on how the particle size of GO and r-GO affects antibacterial activity and that larger GO sheets had a more potent antibacterial activity, probably as larger GO sheets have a greater ability to effectively wrap and isolate bacterial cells preventing further proliferation [58]. These findings contrast with the work of Akhavan et al. [59].

Under acidic conditions,* E. coli *cells were able to reduce GO particles' oxygen containing functional groups as much as 60% in 48 hours, leading to an antibacterial effect [60].

Adding silver particles (Ag) to GO surface increases antibacterial activity [61]. Composite GO-Ag concentrations as low as 10 ppm reduced* E. coli* growth by 99.9%. The interaction of GO-Ag with* E. coli and P. aeruginosa *has been studied, with the authors reporting that bacteriostatic activity is directly proportionate to the concentration of the Ag particles [62].

Gurunathan et al. studied the antibacterial activity of GO and r-GO on* P. aeruginosa* [63].* P. aeruginosa* viability decreased in a dose- and time-dependent manner on contact with GO or r-GO. Dose-dependent antibacterial activity is caused by the production of ROS, leading to cell death.

## 3. Discussion

The interest of bioengineers and surgeons in carbon allotropes has been growing and has been the focus of intensive research in many fields [13, 64–71]. In the 1960s and 1970s, attempts were made to include graphite molecules in vascular grafts and cardiac valve prosthesis [72–75].

Since its production in 2004, graphene has been the tip of the bioengineering spear towards newer and more novel biomaterials. Graphene's unique properties give bioengineering specialists the opportunity to develop new or improve existing materials. In order for a material to be safely deployed and analysed in humans, several characteristics should be investigated: prolonged wear and friction resistance, thrombogenicity, biotoxicity, biocompatibility, interactions with surrounding tissues, and so forth.

The unique structural features of GO give GO excellent biocompatibility, stability, solubility, and drug-bearing capability. Cellular uptake of GO-PEG loaded with chemical drugs has also given promising results [76]. Oligolayer GO is an efficient carrier for delivering medication and genes [77]. Additionally, the reactive groups of GO facilitate chemical interaction and conjugation with an extensive variety of molecules [40, 78–82]. There are already limited data that graphene and especially GO have a great potential in becoming such a drug-eluting delivery system that can reduce endothelial proliferation and restenosis in grafts [22, 26, 27, 33, 83]. A study on the release of anti-inflammatory medication by carbon coating is of relevance to abdominal aortic aneurysm (AAA) repair and the postimplantation syndrome [84]. An interesting approach to drug-eluting stents describes a novel on-demand drug-delivery system based on GO film superimposed on conducting scaffold [84]. This system delivers dexamethasone upon electrical stimulation on the scaffolding, resulting in low-toxicity delivery and controlled dosage. Regarding the incidence of restenosis in carbon-coated stents without the use of protective or drug-eluting films, initial studies are rather ambiguous. A study on the incidence of renal artery restenosis after stenting shows some but nonsignificant benefits of carbon-coated stents when compared to bare steel stents [85]. Coronary carbon-coated stents also showed at least equal results when compared to sirolimus-eluting or bare stents [86–89].

Graphene is the lightest, strongest, and thinnest carbon allotrope and these unique characteristics make graphene a future candidate for construction of stent and endograft scaffolding, but research is necessary to show the number of layers and the allotrope to be used. Several studies exist on how diamond-like carbon (DLC) is already being employed as a stent coating [90–94]. These studies promote this use of DLC by demonstrating slightly better results when compared to control groups or bare stents but fail to show that their result is statistically significant. Two studies report that graphene-covered nitinol stents show improved biocompatibility [26, 27].

As stent material corrosion is already reported, the wear withstanding properties of endomaterials require specific testing [95–97]. Six million cycles of carbon-coated femoral heads do not seem enough to wear the carbon material as graphene and its derivatives are the second strongest material [98]. Therefore, the possibility of wear is low and material corrosion occurs rarely.

Apart from being a building material of stent scaffolds, monolayer graphene may have a future potential as graft material. Graphene sheets are impermeable membranes, to both liquids and gases [99], and could thus be ideal material for decreasing type IV endoleaks. However, this nonporous character of graphene sheets could concomitantly cause reduced patency, as a study suggests that graft material porosity of approximately 60 microns tends to promote early patency [100]. Graphene's one-atom thickness and material elasticity similar to that of PTFE could potentially lead to the development of thinner endografts and delivery systems of smaller diameters, making the catheterization of smaller and more tortuous vessels easier.

Biotoxicity and biocompatibility of graphene and its derivatives are two subjects that have been the focus of many studies [35, 41]. Some authors have reported high* in vitro* cytotoxicity of carbon coatings in different cell lines [34–36, 41]. A smaller number of studies support the biocompatibility of carbon coatings. Some of the latter promote the use of carbon coating without the addition of a protective biofilm [26, 27, 47]. According to other studies, carbon coatings are biocompatible and less cytotoxic only when covered by an additional protective biofilm [22, 40]. This comes to no surprise, as pristine GO without surface functionalization is not stable in biological environments, due to the nonspecific binding of proteins to the surface of GO sheets. The cytotoxic effect of graphene and its derivatives should be studied individually, as it varies based on the molecular structure of the used graphene derivative, the dose, and the time of incubation in a biological environment. The level of potential biotoxicity of nanoobjects, such as graphene sheets or nanoribbons, is strongly affected by the type, amount, and conformation of the attached proteins to the surface of these nanoobjects [101]. This layer of proteins, called* hard corona*, interacts with the biological surroundings of the nanoobject and it can significantly affect the potential cytotoxicity of graphene [37]. Identical graphene derivatives coated with hard coronas made of different proteins exhibit significantly different cytotoxic effect, cell death rates, and ROS generation [101].

Two drawbacks regarding all these studies are that they fail to show a specific threshold of systemic toxicity depending on the dose of released carbon particles and almost all studies are conducted on carbon coatings other than graphene.

Deployed stent grafts, especially in endovascular AAA repair, could become infected, resulting in complications and high postoperative morbidity and mortality [79]. There have been different approaches to making graft materials less prone to bacteria colonization and decreasing the incidence of postoperative graft infection. All published studies on the antibacterial activity of graphene and its derivatives have been conducted with either GO or r-GO. The most common bacteria under investigation are* E. coli* and* S. aureus*. To this moment, no consensus has been reached regarding the antibacterial activity of graphene or graphene-derivative surfaces, but available data suggests that cytotoxicity against bacterial cells varies to some extent on the orientation of the surface particles, a result of the production method used [50, 51, 54, 55, 102]. Another factor altering the antibacterial effect of graphene and its derivatives is the quality of the material. Pristine graphene or graphene produced through epitaxial growth may theoretically have more potent antibacterial activity, but to date there are no studies involving pristine graphene.

Carbon-coated stents release no metal ions into the bloodstream preventing the allergenic or toxic effects of copper, steel, or nitinol stents. Allergic reactions to nickel released from nitinol stents could be significant as toxic effect of Ni ions is already reported in the literature [103].

From all the above, it is obvious that graphene-coated or graphene-built scaffolds have certain advantages over existing bare metal, cobalt-chrome, or nitinol made scaffolds. Without releasing metal ions and by presenting lower thrombogenicity, graphene scaffolds could lead to improved long-term patency and less allergic reactions compared to today's endovascular scaffolds. Metal corrosion, which is evident in other scaffolds after long-term contact with the bloodstream, does not affect graphene scaffolds. The potential of graphene to embed and release molecules could further improve patency by releasing antiplatelet or anticoagulation agents and increase safety by preventing bacterial colonization and preventing in-stent stenosis by releasing cytostatic molecules in well-controlled doses. Particularly, the latter characteristic has only been demonstrated in graphene, while the already used materials (nitinol, cobalt-chrome, and bare steel) do not present with this characteristic.

Some obstacles need to be overcome before deployment of graphene-made materials in humans. Initial data on biostability and biocompatibility are promising, but findings on biotoxicity are ambiguous. Numerous clinical, safety, and regulatory trials are also necessary. However, research to date suggests that graphene and its derivatives could change the face of endovascular surgery.
